# Zinc and Copper Brain Levels and Expression of Neurotransmitter Receptors in Two Rat ASD Models

**DOI:** 10.3389/fnmol.2021.656740

**Published:** 2021-06-29

**Authors:** Elzbieta Zieminska, Anna Ruszczynska, Justyna Augustyniak, Beata Toczylowska, Jerzy W. Lazarewicz

**Affiliations:** ^1^Mossakowski Medical Research Centre, Polish Academy of Sciences, Warsaw, Poland; ^2^Faculty of Chemistry, Biological and Chemical Research Centre, University of Warsaw, Warsaw, Poland; ^3^Nalecz Institute of Biocybernetics and Biomedical Engineering, Polish Academy of Sciences, Warsaw, Poland

**Keywords:** zinc, copper, brain gene expression, autism, animal model

## Abstract

Zinc and copper are important trace elements necessary for the proper functioning of neurons. Impaired zinc and/or copper metabolism and signaling are implicated in many brain diseases, including autism (ASD). In our studies, autistic-like behavior in rat offsprings was induced by application to pregnant mothers valproic acid or thalidomide. Zinc and copper contents were measured in serum and brain structures: hippocampus, cerebral cortex, and cerebellum. Our research shows no interconnections in the particular metal concentrations measured in autistic animal brains and their sera. Based on patient researches, we studied 26 genes belonging to disturbed neurotransmitter pathways. In the same brain regions, we examined the expression of genes encoding proteins of cholinergic, adrenergic, serotonin, and dopamine receptors. In both rats’ ASD models, 17 out of the tested gene expression were decreased. In the cerebellum and cerebral cortex, expression of genes encoding cholinergic, adrenergic, and dopaminergic receptors decreased, whereas in the hippocampus only expression of serotoninergic receptors genes was downregulated. The changes in metals content observed in the rat brain can be secondary phenomena, perhaps elements of mechanisms that compensate for neurotransmission dysfunctions.

## Introduction

Autism spectrum disorders (ASD) are a group of developmental disabilities, which consists mainly of childhood and juvenile autistic disorders, Asperger’s syndrome, and pervasive developmental disorders not otherwise specified. The prevalence of ASD is higher in males (Ramtekkar et al., 2010). The unclear etiology of ASD is thought to be related to the overlap of genetic, epigenetic, and environmental factors (Amal et al., 2020a; Bhandari et al., 2020; Tripathi et al., 2020). The pathogenesis of behavioral disorders observed in ASD is also the subject of hypotheses. The authors point out, among others, the presumed role of oxidative stress, disorders of neurotransmission, and also disturbances in trace metal metabolism, primarily zinc and copper in the brain (Bjorklund, 2013; Paval, 2017; Bozzi et al., 2018; Garbarino et al., 2019; Behl et al., 2020; Bjorklund et al., 2020).

Zinc and copper are trace elements necessary for the proper functioning of cells, including brain cells. Many brain diseases, including autism, are characterized by impaired zinc and/or copper metabolism and signaling (Bjorklund, 2013; Scheiber et al., 2014; Portbury and Adlard, 2017; Ackerman and Chang, 2018). Deficiencies of Zn and Cu or excessive burden of the mother’s body of toxic metals can be one of many environmental factors triggering the occurrence of ASD in offspring (Grabrucker et al., 2016; Bolte et al., 2019). Moreover, zinc deficiency and/or impaired Cu/Zn ratio in blood, hair, and nails have been reported in children with autism (Faber et al., 2009; Lakshmi Priya and Geetha, 2011; Craciun et al., 2016). Despite this, changes in metals content on the periphery do not necessarily indicate the presence of analogous disorders in brain cells. As there is insufficient brain data for ASD patients, we assume that research using animal models of autism can shed light on these problems.

Behavioral symptoms in patients with ASD are the result of disorders in chemical neurotransmission in the brain. Many researchers indicated a relationship between disorders of zinc and copper homeostasis and abnormalities in chemical brain neurotransmission that may be associated with ASD pathogenesis (Moroni et al., 2008; Li et al., 2015; Szewczyk et al., 2017; Lutsenko et al., 2019). Therefore, an interesting complement to the results of studies on the levels of zinc and copper in different regions of the brain in animal models of autism would be to examine in the same material the expression of genes encoding proteins of selected types of receptors, other than glutamatergic that has been already studied (Arons et al., 2016; Ha et al., 2018; Hagmeyer et al., 2018). To our knowledge, there are no results available for such studies on patients and animal models based on administering development toxins to pregnant female rats. Among them, ASD models based on the exposure of pregnant female rats to teratogenic drugs such as valproic acid (VPA) or thalidomide (THAL) are widely used.

There are reports of the influence of sex on biological processes in animals (Khaliulin et al., 2020) hence, the present study aimed to investigate serum zinc and copper concentrations in juvenile 1-month-old rats, males and females, in ASD models induced by VPA or THAL, compared to age and gender matched control groups. Then, we examined the content of these metals and the expression of acetylcholine, adrenergic, serotonin, and dopamine receptor genes in the frontal cortex, hippocampus, and cerebellum of rats from both experimental models. In our research, we focused on checking Zn and Cu imbalance in rats in ASD models resembling the changes described in patients. Possible effects of disturbances in rare metal homeostasis on neurotransmission in the brain have been suggested. Therefore, we attempted to detect changes in the expression of genes encoding receptors for various neurotransmitters that could be linked to metallomics results. We searched for similarities or inconsistencies in the changes in both tested parameters between the study groups, and we tried to make comparisons of these results with data from the study of patients with ASD. We expected that this could shed new light on the role of Zn and Cu in the pathogenesis of ASD, and the suitability of our rat models for such studies.

## Materials and Methods

### Animal Models of Autism

The procedure of inducing two chemical teratogenic models of autism in rats was performed exactly as previously described (Zieminska et al., 2018). Female rats on the 11th day of gestation were fed by intra-gastric tube one dose of VPA (800 mg/kg b.w. mixed with 1 ml saline solution) or THAL (500 mg/kg b.w. mixed with vegetable oil). Control animals were treated with 1 ml of a mixture of oil and saline, 1:1 v/v (Kolozsi et al., 2009; Narita et al., 2010). The ultrasonic vocalization test was carried out on PND 9 rats from all experimental and control groups. The results, i.e., a significantly reduced level of ultrasonic vocalization emitted by pups from the VPA- and THAL-treated groups after separation from the mothers, which is considered to be a reliable indicator of pathology similar to autism in rats, did not differ from those described previously (Zieminska et al., 2018).

The studies were performed using the 35-day-old Wistar rats (Cmd: (WI)WU) of both sexes. Rats were bred in the Animal Colony of the Mossakowski Medical Research Centre, Polish Academy of Sciences in Warsaw. The animals were supplied water and fed *ad libitum*. They were kept in cages on a twelve-hour dark-light cycle at room temperature with a ∼60% humidity. Newborn rats were bred along with their mothers in individual litters. After 21 days from birth, the pups were separated from their mothers and divided into study groups: control, VPA, and THAL, 3–4 individuals of the same sex per cage. For each test group in our study, the animals came from four litters. All procedures involving animals were conducted in accordance with Directive 2010/63/EU on the protection of animals used for scientific purposes and with adherence to the national regulations. They were approved by the Fourth Local Ethical Committee in Warsaw, resolution No. 43/2015 on May 22, 2015.

### Brain Regions and Serum Used in the Experiments

The 35-day-old rats from the experimental and control groups were sacrificed by decapitation for genetic studies and for determination of metal content in the brain. Three areas of the brain were selected for our study: the frontal cortex, part of the cerebral cortex (CC), the hippocampus (HPC), and the cerebellum (CE). The CC, CE, and HPC samples used for determination of metal content were cleaned of meanings and vessels and frozen separately at −80°C until use. The number of animals used in this experiment was: 21 (6F + 15M) in the control group; 21 (9F + 12M) in the VPA group, and 18 (6F + 12M) in the THAL group.

Blood samples were collected on a clot from the hearts of the animals just before brain perfusion (brains prepared in this way were used in another experiment not described in this manuscript). Samples were then centrifuged at 265 × *g* for 10 min to obtain serum. Serum samples were frozen at −20°C until use. Control samples were collected from 37 animals (18F + 19M); 35 (24F + 11M) from VPA-treated rats and 22 (10F + 12M) from THAL-treated animals.

**FIGURE 1:**
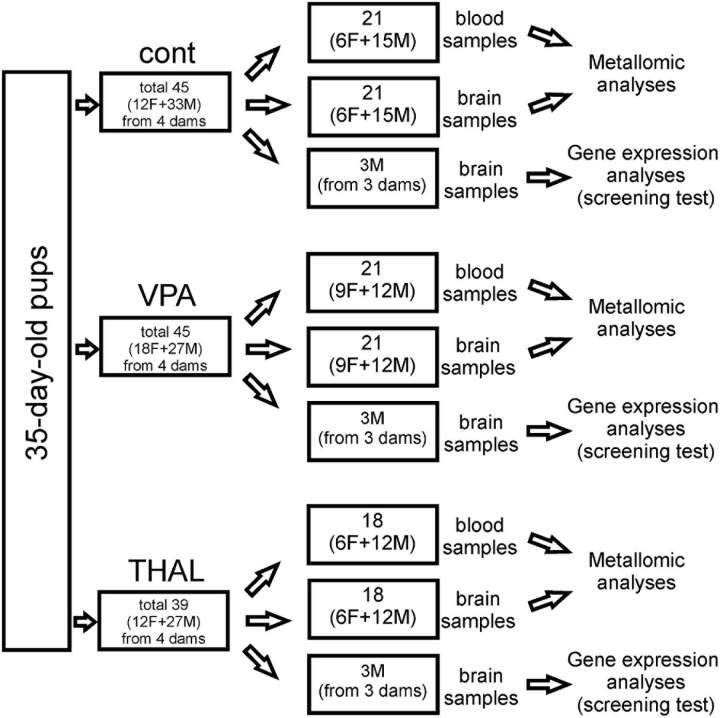
Cont – control group, VPA – valproate group, THAL – thalidomide group, F – female, M – male.

CC, CE, and HPC samples for genetic screening were isolated from the rat brain using the method described by Spijker (2011). In each group, these samples were collected from 3 male rats representing 3 independent experiments. Pups come from 3 separate litters, whose mothers received VPA or THAL during pregnancy or from control litters. Brain tissue was isolated from randomly selected control subjects and from those rats from the experimental groups that had significantly reduced levels of ultrasonic vocalization emitted by pups at PND 9 after separation from their mothers. Each part of the brain was placed in fenezol solution, a component of the Total RNA Mini Kit (A&A Biotechnology), and stored at −25°C until RNA isolation was carried out. The number of animals used in all experiments is presented in Figure 1.

### Determination of Zinc and Copper

Serum samples with a volume of 30 μl were diluted 50 times with 1% nitric acid just before determining the total metal content. Brain region samples were digested in the microwave system Ultra Wave (Milestone, Italy) with closed Teflon vessels. Samples between 0.050 and 0.400 g were digested in 1 ml nitric acid with the addition of 30% hydrogen peroxide (0.1 ml) under the following conditions: 15 min up to 220°C and 10 min at 220°C. Blanks were prepared according to the same procedure and digested in each cycle. Measured results by ICP MS were subtracted from the results for the samples. Cooled digests were diluted with water up to 5 ml and analyzed by quadrupole mass spectrometry with inductively coupled plasma ionization (ICP-MS). Determination of total content of Cu and Zn was achieved using ICP-MS (Nexion 300D, Perkin Elmer, United States) equipped with a liquid sample introduction system consisting of a quartz nebulizer (Meinhard) and a quartz cyclonic spray chamber. The spectrometer parameters were optimized daily in order to obtain the maximal sensitivity and stability whilst the lowest level of oxides and double-charged ions. The ICP-MS conditions were as followed: a radio frequency power RF 1350 W, flow rate of plasma gas 15 l/min, auxiliary gas 1.2 l/min, and nebulizer gas 0.86 l/min, dwell time 100 ms and monitored isotopes ^63^Cu and ^66^Zn. Five point of calibration curve with correlation coefficient R2 = 0.9999 was achieved in the range from 0.30 to 30 μM and the procedure was validated by the analysis of certified reference material MODAS-4 obtained from LGC Standards (Poland). Recovery for Zn and Cu was 90 and 110%, respectively. Each sample was measured in triplicates and precision didn’t exceed 5%. The limit of quantification (LOQ) calculated for the results obtained from blanks undergoing the same procedure as samples was 0.04 μM for ^66^Zn and for 0.01 μM^63^Cu (LOQ = mean + 6 SD). The total content was standardized according to the wet weight of samples. Data were statistically analyzed using the univariate one-way and two-way ANOVA tests followed by Holm-Sidak corrections (SigmaPlot v. 12.5, Systat Software, Inc.). The *p*-value lower than 0.05 was considered significant.

### Gene Expression Analysis (RT-qPCR)

In our previous works (Lenart et al., 2020b; Toczylowska et al., 2020), we did not find significant statistical differences between males and females within the study group. Therefore, we decided to perform our first screening test of genes encoding proteins of selected receptors, involved in the pathogenesis of ASD, on males, in which we observed slightly greater changes than in females.

#### RNA Isolation

RNA was isolated from rat brain tissue samples exactly as described previously (Lenart et al., 2020a,b). Homogenates from particular regions of 3 animals were analyzed separately. RNA from each part of the brain of each animal was isolated in two independent replicates, in addition RT-qPCR reactions were performed in 2 technical replicates. RNA isolation was performed using the Total RNA Mini Kit (A&A Biotechnology, Gdynia, Poland) according to the manufacturer’s instructions. Genomic DNA was eliminated by DNase treatment using a clean-up RNA concentrator kit (A&A Biotechnology, Gdynia, Poland). The concentration of RNA was determined by measuring the absorbance at 260 nm with a NanoDrop ND-1000 spectrophotometer (Thermo Fisher Scientific, Waltham, MA, United States), and its purity was assessed by calculating the 260 nm/280 nm absorbance ratio.

#### RNA Quality Improvement

RNA integrity was assessed by the 3′/5′ integrity assay. Two assays were designed along the length of the succinate dehydrogenase complex flavoprotein subunit A (Sdha) cDNA. One is located close to the 3′ UTR (Sdha 3′), and the second is approximately 1 kb upstream, close to the 5′ end (Sdha 5′). cDNA was generated using reverse transcription from an oligo-dT primer from the TranScriba kit (A&A Biotechnology, Gdynia, Poland). Following amplification by RT-qPCR, the products from each assay were quantified, and the differences between 3 and 5′Cq, ΔCq = |3′Cq–5′Cq| were calculated. If the value of ΔCq < 1 RNA, then degradation is considered minimal and will likely have a minimal effect on gene expression results. ΔCq > 1 suggests that the RNA integrity may compromise gene expression results, and such samples were removed from the analysis. The primers for the integrity test were designed using the PrimerQuest (eu.idtdna.com): Sdha5′ (Sdha5′F TGGCTTTCACTTCTCTGTTGG, Sdha5′R TGGGTAGAAATCGCGTCTGA); Sdha3′ (Sdha3′F AAGAAGC CATTTGCGGAACA, Sdha3′R GTAACCTTCCCAGTCT TGGTG). Each primer’s specificity was checked using Primer-BLAST software^1^.

#### Reference Gene Validation

Reference gene validation was performed with NormFinder software in accordance with the principles set out in our previous publications (Lenart et al., 2017; Augustyniak et al., 2019). Actb (actin), B2m (beta-2-microglobulin), Gapdh (glyceraldehyde-3-phosphate dehydrogenase), Gusb (beta-glucuronidase), Hmbs (porphobilinogen deaminase), Hprt1 (hypoxanthine-guanine phosphoribosyltransferase), Rpl13a (60S ribosomal protein L13a), Sdha (succinate dehydrogenase [ubiquinone] flavoprotein subunit), Tbp (TATA-box-binding protein), Ppia (peptidyl-prolyl *cis-trans* isomerase A), Ubc (polyubiquitin-C precursor), and Ywhaz (14-3-3 protein zeta/delta) were chosen as reference housekeeping genes. The custom gene panel (Bio-Rad, Hercules, CA, United States) provided the primers for these reference genes.

#### RT-qPCR Reaction

Samples taken from three brain regions of rats treated with VPA (*n* = 3) and rats in the control group (*n* = 3) were analyzed using RT-qPCR. The RT-qPCR reactions were replicated three times for each sample. Expression profiling of the custom gene panel (Bio-Rad, Hercules, CA, United States) was performed using 10 ng of cDNA per reaction. Each RT-qPCR contained 1 μl of cDNA sample and 10 μl of SsoAdvanced Universal SYBR Supermix (Bio-Rad, Hercules, CA, United States in a total volume of 20 μl. Reactions were performed in three technically identical replicates. RT-qPCR was performed using the LightCycler 96 (Roche Diagnostics GmbH) through the following steps: initial denaturation step at 95°C for 2 min, followed by 40 cycles of denaturation at 95°C for 5 s and annealing/elongation at 60°C for 30 s. Specificity of target amplification was confirmed by melting curve analysis. Every assay contained the positive RT-qPCR control, the reverse transcription (RT) control, the DNA contamination control, the RNA quality control and the panel of reference genes. The results were analyzed using PrimePCR Analysis Software (Bio-Rad, Hercules, CA). The data in Table 1 are presented as fold changes in relative normalized expression, assuming >4-fold as upregulation and <−4-fold as downregulation.

**TABLE 1:** Genes up-regulated and down-regulated in the cerebral cortex (CC), cerebellum (CE), and hippocampus (HPC) of rats in the VPA-treated group.

Brain region	Target	Samples	Average of relative normalized gene expression	SEM	Regulation FC (*fold changes*)	Compared to Regulation Threshold (FC < −4 down-regulated, FC > 4 up-regulated)
**CC**	*Adra1d*	Control	1.00	0.22	7.12	Up-regulated
		VPA	7.12	9.34		
	*Adra2a*	Control	1.00	0.12	59.61	Up-regulated
		VPA	59.60	78.12		
	*Chrna5*	Control	1.00	0.15	4.07	Up-regulated
		VPA	4.07	5.33		
	*Chrna7*	Control	1.00	0.15	119.63	Up-regulated
		VPA	119.63	156.9		
	*Chrne*	Control	1.00	0.13	−4.44	Down-regulated
		VPA	0.23	0.30		
	*Drd2*	Control	1.00	0.19	−5.33	Down-regulated
		VPA	0.19	0.30		
**CE**	*Adra1a*	Control	1.00	0.79	−5.20	Down-regulated
		VPA	0.19	0.18		
	*Adra1d*	Control	1.00	1.09	−9.52	Down-regulated
		VPA	0.11	0.10		
	*Adcy7*	Control	1.00	0.48	−4.21	Down-regulated
		VPA	0.24	0.36		
	*Chrm1*	Control	1.00	2.39	−18.70	Down-regulated
		VPA	0.05	0.05		
	*Chrm4*	Control	1.00	1.94	−8.29	Down-regulated
		VPA	0.12	0.06		
	*Chrna5*	Control	1.00	2.69	−13.32	Down-regulated
		VPA	0.08	0.03		
	*Chrna7*	Control	1.00	1.02	−5.15	Down-regulated
		VPA	0.19	0.12		
	*Drd1a*	Control	1.00	0.27	−7.49	Down-regulated
		VPA	0.13	0.06		
**HPC**	*Htr1b*	Control	1.00	0.66	−11.23	Down-regulated
		VPA	0.09	0.11		
	*Htr1d*	Control	1.00	0.47	−4.29	Down-regulated
		VPA	0.23	0.13		
	*Htr3a*	Control	1.00	0.66	−11.81	Down-regulated
		VPA	0.09	0.11		

## Results

### Metallomic Analyses

**FIGURE 2:**
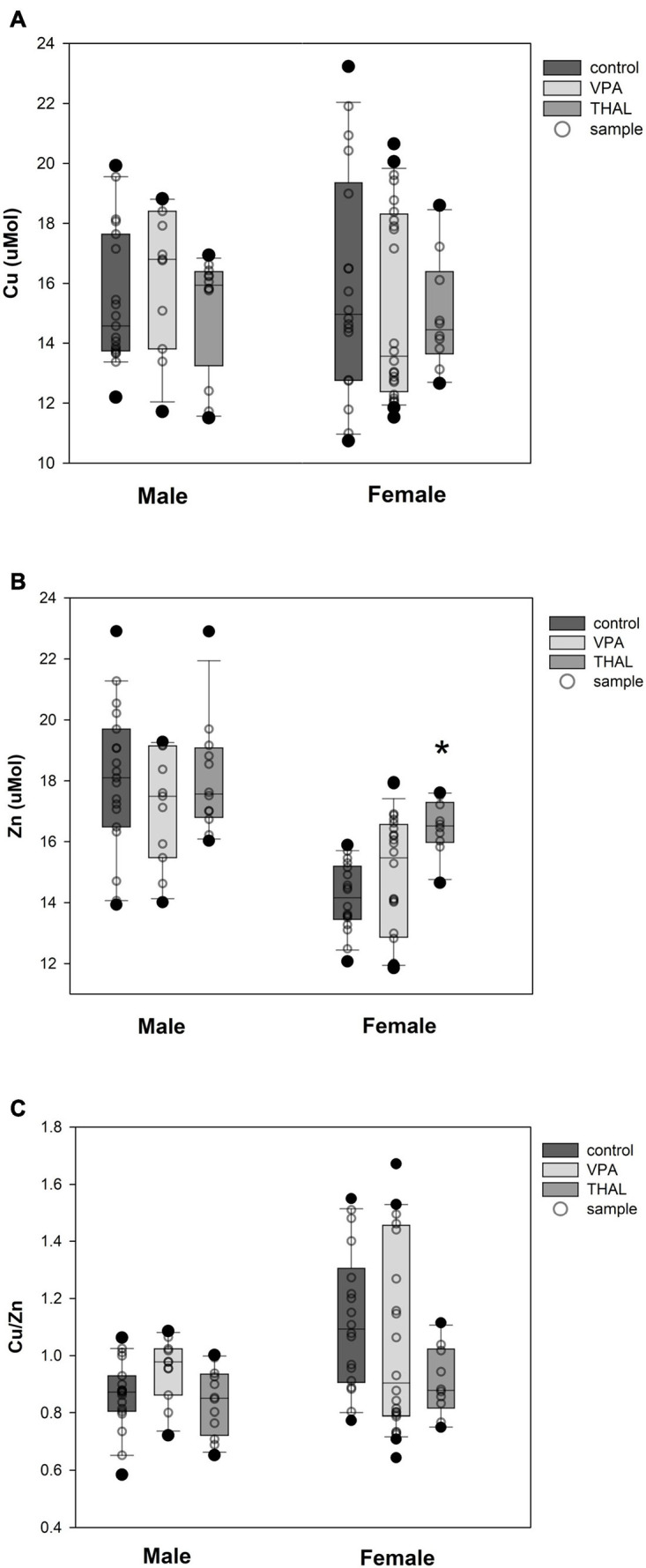
Box and Whiskers plot of changes in Cu **(A)**, Zn **(B)**, and Cu/Zn **(C)** levels in rats’ serum measured using the ICP-MS method. The obtained results are expressed in μM concentration **(A,B)**. Number of samples: *n* = 37 (18F + 19M) – control group, *n* = 35 (24F + 11M) – VPA-treated rats, and *n* = 22 (10F + 12M) – THAL-treated animals; F – female; M – male rats; (*) represents results statistically significant vs. control, *p* < 0.05, one-way ANOVA followed by Holm-Sidak corrections; box containing 50% of results that fell in the range between 25% (bottom line) and 75% (top line) – IQR (InterQuartile Range), (–) median value, whiskers – min and max values, (∘) – outliers (±1.5 × IQR), (∘) – individual sample.

The results of measurements (means ± SEM) of copper and zinc concentrations, as well as the ratio of copper to zinc in the blood serum of male and female rats from the VPA and THAL groups compared to the control are shown in Figures 2A–C. In the groups of males that were treated with VPA or THAL during the fetal period, we found no differences in Cu and Zn concentrations, and in the ratio of Cu to Zn concentrations, compared to the appropriate controls. In contrast, among females, a statistically significant increase in zinc concentration was detected in the THAL group (*p* = 0.006). The mean Zn concentration in the control group was 14.2 ± 0.4 and in the THAL group 16.5 ± 0.6.

**FIGURE 3:**
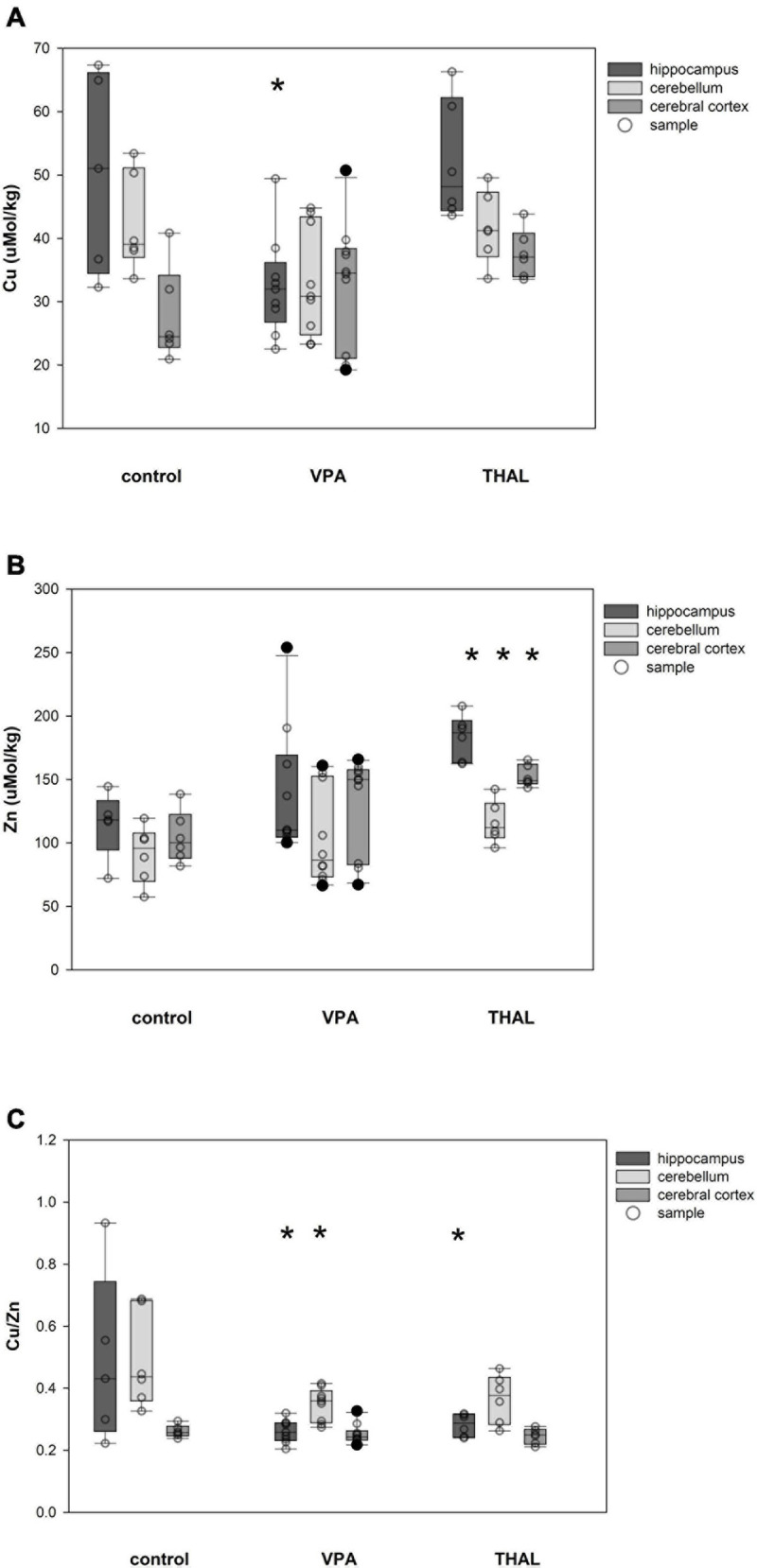
Box and Whiskers plot of changes in Cu **(A)**, Zn **(B)**, and Cu/Zn **(C)** levels in female rats in parts of brain: hippocampus, cerebellum and cerebral cortex, measured using the ICP-MS method. The obtained results are expressed in concentration μM/kg wet tissue **(A,B)**. The number of animals used in this experiment was: *n* = 6 – control group, *n* = 9 – VPA-treated rats, and *n* = 6 – THAL-treated animals; (*) represents results statistically significant vs. control, *p* < 0.05, two-way ANOVA followed by Holm-Sidak corrections; box containing 50% of results that fell in the range between 25% (bottom line) and 75% (top line) – IQR (InterQuartile Range), (•) median value, whiskers – min and max values, (∘) – outliers (±1.5 × IQR), (–) – individual sample.

Cu and Zn content as well as Cu/Zn ratio in homogenates of the HPC, CE, and CC of females in the control and both experimental groups are shown in Figures 3A–C. Significant differences in Cu concentration were observed for the VPA group in HPC (*p* = 0.002). The mean Cu concentrations were 50.5 ± 4.3 for the control group and 32.5 ± 3.2 μM/kg for the VPA group. Significant differences were observed in females in Zn concentration in HPC (*p* < 0.0001), CE (*p* = 0.031), and CC (*p* < 0.0001). Mean Zn concentrations for control group were 114.9 ± 8.6; 91.1 ± 7.9, and 104.7 ± 7.9 μM/kg for HPC, CE, and CC, respectively. Mean Zn concentration for the THAL group were 183.5 ± 7.9, 116.3 ± 7.9, and 152.8 ± 7.9 μM/kg for HPC, CE, and CC, respectively. Cu/Zn ratio was significant different in HPC (*p* < 0.013). Mean ratio for the control group for HPC was 0.49 ± 0.06. For the THAL group this value was 0.28 ± 0.05. Cu/Zn ratio was significant different in HPC (*p* < 0.0001) and CE (*p* = 0.019). Mean ratio for the control group for HPC and CE was for both regions 0.49 ± 0.05. For the VPA group for these regions, these values were 0.26 ± 0.04 and 0.35 ± 0.04.

**FIGURE 4:**
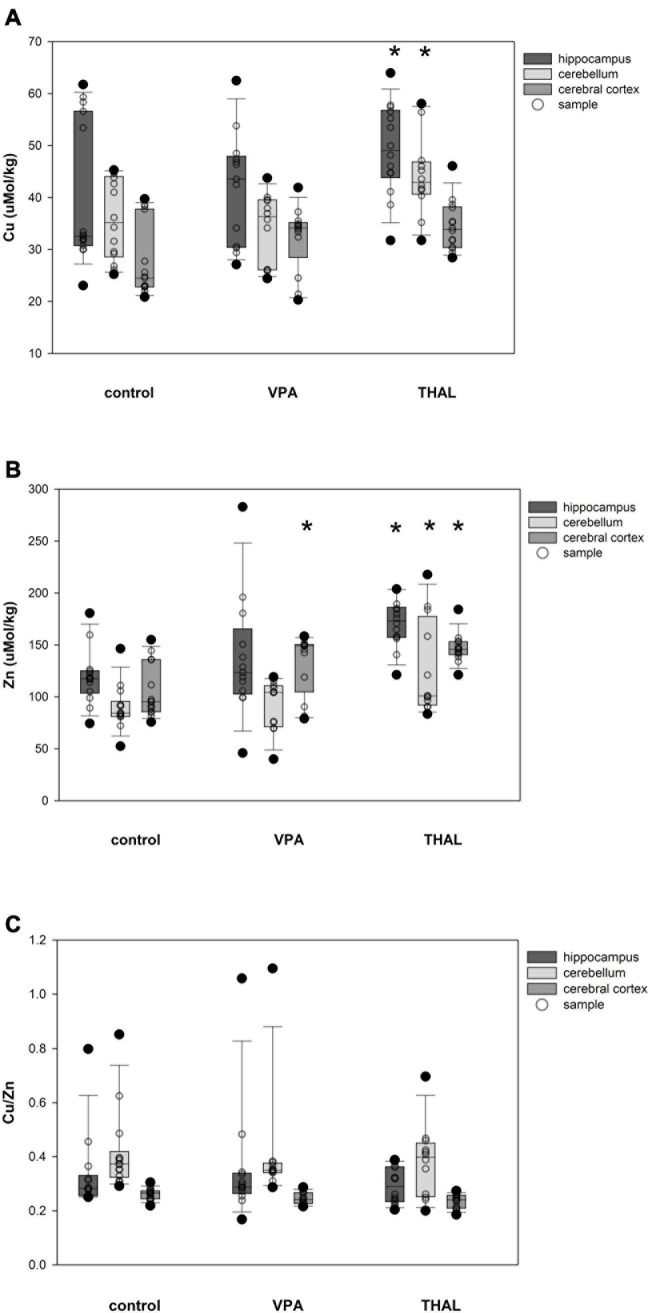
Box and Whiskers plot of changes in Cu **(A)**, Zn **(B),** and Cu/Zn **(C)** levels in male rats in parts of brain: hippocampus, cerebellum and cerebral cortex, measured using the ICP-MS method. The obtained results are expressed in concentration μM/kg wet tissue **(A,B)**. The number of animals used in this experiment was: *n* = 15 – control group, *n* = 12 – VPA-treated rats, and *n* = 12 – THAL-treated animals; (*) represents results statistically significant vs. control, *p* < 0.05, two-way ANOVA followed by Holm-Sidak corrections; box containing 50% of results that fell in the range between 25% (bottom line) and 75% (top line) – IQR (InterQuartile Range), (–) median value, whiskers – min and max values, (•) – outliers (±1.5 × IQR), (∘) – individual sample.

The content of Cu and Zn and the ratio of Cu/Zn in HPC, CE and CC homogenates in males in the control and both experimental groups are shown in Figures 4A–C. Only in the THAL group Cu concentration was significantly different in HPC (*p* < 0.001) and CE (*p* = 0.017) compared to the control. Mean concentrations for the control group in HPC and CE were 39.87 ± 2.22 and 35.83 ± 2.3, respectively. Concentrations in the THAL group for these regions were 51.01 ± 2.49 and 44.13 ± 2.49, respectively. Significant differences were observed in Zn concentration in HPC (*p* < 0.0001), CE (*p* < 0.0001), and CC (*p* < 0.0001). Mean Zn concentrations for the control group for HPC, CE, and CC, were 119.2 ± 7.2; 90.1 ± 7.2, and 104.8 ± 6.9 μM/kg, respectively. Mean Zn concentrations for THAL group were 173.9 ± 7.7; 127.7 ± 7.7, and 145.9 ± 7.7 μM/kg for HPC, CE, and CC, respectively. In males, in all examined brain regions, there were no differences in the Cu/Zn ratio in both the VPA and THAL groups compared to the controls group.

### Gene Expression Analyses

In the present study the expression of 24 genes encoding acetylcholine, adrenergic, serotonin and dopamine receptor proteins, and two additional proteins involved in adrenergic signaling were examined in the HPC, CE, and CC of male rats, representing the control, VPA, and THAL groups. The Electronic Supplementary Material contains a list of tested genes (Supplementary Table 1) and the results of the *in silico* analysis showing networks of predicted functional interactions of cholinergic, adrenergic, serotoninergic and dopaminergic genes. There is also extensive information available on the functions assigned to these genes (Supplementary Figures 1–5).

The results of gene analysis (the extent and direction of changes in the expression of these genes) carried out separately for individual brain regions of rats from the VPA and THAL groups compared to the control are presented in the Tables 1, 2 and Supplementary Figure 6.

**TABLE 2:** Genes up-regulated and down-regulated in the cerebral cortex (CC), cerebellum (CE), and hippocampus (HPC) of rats in the THAL-treated group.

Brain region	Target	Samples	Average of relative normalized gene expression	SEM	Regulation FC (*fold changes*)	Compared to Regulation Threshold (FC < −4 down-regulated, FC > 4 up-regulated)
**CC**	*Adra2a*	Control	1.00	0.12	5.02	Up-regulated
		THAL	5.02	1.05		
	*Adrb3*	Control	1.00	0.15	−28.63	Down-regulated
		THAL	0.04	0.02		
	*Adrbk1*	Control	1.00	0.19	−5.98	Down-regulated
		THAL	0.17	0.04		
	*Chrm5*	Control	1.00	0.45	−7.49	Down-regulated
		THAL	0.13	0.03		
	*Chrna7*	Control	1.00	0.15	5.66	Up-regulated
		THAL	5.66	0.73		
	*Chrne*	Control	1.00	0.13	−67.59	Down-regulated
		THAL	0.02	0.01		
	*Drd2*	Control	1.00	0.19	−15.81	Down-regulated
		THAL	0.06	0.01		
	*Drd5*	Control	1.00	0.12	−26.94	Down-regulated
		THAL	0.04	0.01		
**CE**	*Adra1d*	Control	1.00	1.09	−9.71	Down-regulated
		THAL	0.10	0.18		
	*Chrm1*	Control	1.00	2.39	−11.70	Down-regulated
		THAL	0.09	0.13		
	*Chrm4*	Control	1.00	1.94	−12.13	Down-regulated
		THAL	0.13	0.07		
	*Chrna4*	Control	1.00	0.85	−7.74	Down-regulated
		THAL	0.13	0.26		
	*Chrna7*	Control	1.00	1.02	−6.79	Down-regulated
		THAL	0.15	0.24		
	*Drd1a*	Control	1.00	1.09	−15.35	Down-regulated
		THAL	0.19	0.53		
	*Htr1f*	Control	1.00	0.27	−4.66	Down-regulated
		THAL	1.07	0.11		
**HPC**	*Htr1b*	Control	1.00	1.67	−10.89	Down-regulated
		THAL	0.09	0.12		
	*Htr1d*	Control	1.00	1.19	−55.24	Down-regulated
		THAL	0.02	0.04		
	*Htr3a*	Control	1.00	1.67	−9.71	Down-regulated
		THAL	0.10	0.14		

Changes in the expression of genes encoding cholinergic muscarinic and nicotinic receptor proteins (Tables 1, 2) both in the VPA and THAL groups, occurred only in the CE and CC, but they were not found in the HPC. In the VPA group, there was a decrease in the expression of *Chrm1* and *Chrm4* genes encoding corresponding muscarinic receptors as well as *Chrna5* and *Chrna7* genes of nicotinic receptors in the cerebellum. In contrast, the expression of *Chrna5* and *Chrna7* in the frontal cortex increased, the latter by 120 times, while *Chrne* expression decreased. In the THAL group, the expression of *Chrm1, Chrm4*, and also of *Chrna4* and *Chrna7* in the cerebellum decreased, whereas in the frontal cortex there was a decrease in Chrm5 expression and a 67-fold reduction in nicotinic *Chrne* gene expression. In this group, the increase in expression concerned only the *Chrna7* gene in the frontal cortex.

Also, changes in the expression of adrenergic genes (Tables 1, 2) were not observed in the HPC in both experimental groups. In the VPA group, the expression of *Adra1a* and *Adra1d* alpha adrenergic receptors and of adenylate cyclase 7 gene *Adcy7*, which is involved in adrenergic signaling, decreased in the CE. In turn the expression of the alpha adrenergic genes *Adra1d* and particularly *Adra2a* in the CC increased, the latter almost 60-fold. In the THAL group, we found only decreases in the expression of the *Adra1d* gene in the CE, and in the CC, of the beta adrenergic *Adrb3* as well as of the *Adrbk1* gene encoding the G protein-coupled receptor kinase 2/beta-adrenergic receptor kinase 1, also involved in regulation of adrenergic signaling.

A specific pattern of changes in the expression of serotonergic genes was observed (Tables 1, 2): they concerned almost exclusively the HPC and in both experimental groups consisted solely in the reduction of *Htr1b, Htr1d*, and *Htr3a* expression. The only exception was a decrease in *Htr1f* expression in the CE in the THAL group.

In both experimental groups, only decreases in dopaminergic gene expression were noted (Tables 1, 2). This applies to genes *Drd1* in the CE and *Drd2* in the CC. In addition, in the THAL group there was a significant decrease in *Drd5* expression in the CC.

## Discussion

We chose two commonly used chemical models of autism for our research. VPA and THAL were administered to pregnant mothers during periods of particular fetal sensitivity to factors inducing disorders of brain development (Narita et al., 2010). Behavioral symptoms resembling autistic disorders as well as neurochemical changes were observed in the offspring (Rodier et al., 1996; Schneider and Przewlocki, 2005; Sandhya et al., 2012; Zieminska et al., 2018). This justifies the use of animal models in studies of the pathogenesis of autism. In contrast to genetic models (Penagarikano et al., 2011; Angelakos et al., 2019) based on studying the effects of a single gene dysfunction, these models allow analyzing multi-gene changes focusing on their influence on behavior and can reveal new molecular mechanisms underlying autism. Based on our previous results (Lenart et al., 2020a; Toczylowska et al., 2020), we can conclude that these two models used in our study are not identical, although induce behavioral symptoms similar to autism. In our opinion, both of them are good candidates for models of idiopathic autism. In our research, we used a standardized rodent diet that is balanced in terms of all nutrients, including metals. We did not change their food both *in utero* and after birth. This allowed us to track changes in the distribution of metals in the offspring after a single administration of VPA and THAL to pregnant females compared to the control group. In our studies, we did not examine the effect of a metal deficiency (Grabrucker et al., 2017) or excess (Amal et al., 2020b) on the development of an autistic phenotype, but we did examine how the administration of toxins (VPA or THAL) that induced autism-like behavior influenced the distribution of metals in the rat brain. The subject of the study of this work were three regions of the brain: CC, HPC, and CE, because their developmental disorders are particularly strongly associated with ASD pathogenesis (Varghese et al., 2017; Bruchhage et al., 2018; Sun et al., 2019). In previous studies, also done on the THAL- and/or VPA-induced rat models of autism we showed changes in the content of neuroactive amino acids in the hippocampus (Zieminska et al., 2018), and then we did the metabolomic profiles of this area of the brain (Toczylowska et al., 2020). In addition, we investigated changes in SNAP-25 protein expression and in the expression of glutamatergic and GABAergic genes in the cerebral cortex, hippocampus and cerebellum (Lenart et al., 2020a,b).

The starting point for our studies was abnormal Zn and Cu levels in serum and peripheral cells in patients with ASD, i.e., Zn deficit and an increase in the Cu/Zn ratio presented by other research groups (Faber et al., 2009; Russo and Devito, 2011; Bjorklund, 2013; Paval, 2017; Bozzi et al., 2018; Garbarino et al., 2019; Bjorklund et al., 2020). It is also known that Zn and Cu homeostasis disorders cause changes in synaptic neurotransmission in the brain (Moroni et al., 2008; Li et al., 2015; Szewczyk et al., 2017; Lutsenko et al., 2019), so it can be hypothesized that this way these metals can participate in the mechanism of behavioral disorders in patients with ASD. In our present study, we checked whether changes in serum Zn and Cu levels similar to those observed in patients with ASD are revealed in two rat ASD models. Due to the lack of literature data on the levels of Zn and Cu in the brain in autistic patients, we also examined the content of these metals in three rat brain regions in the studied groups. Both VPA and THAL groups have been shown, in our previous studies, to exhibit autism-like behavioral deficits (Zieminska et al., 2018). Given the mutual interaction between Zn and Cu, and neurotransmission activity, we were also looking for analogies in both experimental groups in those metal levels and expression of genes encoding cholinergic, adrenergic, serotoninergic, and dopaminergic receptor proteins. In discussing the results obtained on ASD animal models, we pay special attention to their comparability with clinical observations and their usefulness for verifying the hypothesis about the interrelationship between Zn and Cu imbalance and neurotransmission disorders in ASD.

The analysis of serum copper and zinc concentrations and the content of these metals in the brain in both rat ASD models demonstrated an almost complete absence of their changes in the VPA model, while in the THAL model we observed an increase in serum Zn concentration in females. An increase in both Zn and Cu content in the examined areas of the brain, especially in males was observed. We did not find changes in the serum Cu/Zn ratio in any of the experimental groups of both sexes as well as in any brain structures of those groups in males. Summarizing the results of our current study, we can state the inconsistency of the results of metal levels in serum of both rat ASD models with the corresponding results obtained from studies of autistic patients. We do not believe that these differences can be attributed to the age mismatch of the studied rats to the age groups of children with ASD, in whom disorders of the zinc and copper levels were described. Significantly lower levels of whole blood Zn and higher Cu/Zn ratio compared to control were observed in the ASD children at the age of about 6 (Craciun et al., 2016), and a significant increase in Cu content with large variations in Zn content in hair and nails was reported in autistic children aged 4–12 (Lakshmi Priya and Geetha, 2011). The 1-month-old rats used in our studies fit well with these age groups of children, as a 6-week-old rat is considered to be developmentally equivalent to a 12–13-year-old child [www.ratbehavior.org/RatYears.htm]. These comparisons of the age of children and juvenile rats must be considered when we compare the results of studies on autistic children with those obtained in ASD rat models, both metallomic and genetic. Furthermore, it should be kept in mind that ASD is a developmental disease and that metallomic profiles and patterns of gene expression change may change with the age of patients and animal models (Kartawy et al., 2020).

Our research also revealed differences in these results between the VPA and THAL groups, in which identical behavioral disorders were observed. These results collectively may indicate the low importance of changes in Zn and Cu homeostasis in the pathogenesis of autism-like behavioral disorders and the secondary nature of these changes. It is worth noting that a reduction in zinc content and/or an increase in peripheral copper levels have been observed in ASD patients from areas where nutritional deficiencies and environmental poisoning are likely (Faber et al., 2009; Craciun et al., 2016), while no disturbances in blood zinc levels have been reported in children with ASD originating in Ireland, where dietary deficiencies are less likely (Sweetman et al., 2019). In our study, the rats also received a standard, environmental toxin-free diet that contained micronutrients. This may explain why in both experimental groups in rats we did not find changes in Zn and Cu levels similar to those described in several publications in autistic patients. The verification of this hypothetical explanation requires further studies on the rat VPA and THAL models, supplemented by the study of the effect of a Zn-depleted diet enriched in Cu. There are no studies by other researchers, to our knowledge, that explain the mechanism of differences between VPA and THAL groups, in the levels of Zn and Cu in the serum and the brain regions studied, and why the content of both examined metals in the brains of the THAL group increases simultaneously. There may be some specific differences in neurodevelopmental disorders caused by both teratogenic drugs, which would result in different metal levels. This could be an effect of the induction of apometallothionein, an intracellular protein binding both Zn and Cu, higher in THAL rats (Sutherland and Stillman, 2014). This temporary explanation must wait for experimental confirmation. In summary, the results of our metallomic studies conducted on rat models of ASD induced by application of VPA or THAL to pregnant mothers indicate limited usefulness of these models for the reproduction of changes in Zn and Cu levels observed in autistic patients. It seems that such studies should be supplemented by examining the effect of modifying the content of tested metals in the diet.

The next goal of our research was to examine whether the expression of cholinergic, adrenergic, serotonin, and dopaminergic systems receptors are impaired in our rat models of autism. We conducted screening of mRNA levels of selected genes (Supplementary Table 1) that affect the structure of receptors from the above-mentioned systems in three brain regions where we previously tested Zn and Cu levels (Tables 1, 2). As described in the literature (Marotta et al., 2020) and presented in the Supplementary Material (Supplementary Tables 2, 3), receptors of these neurotransmitter systems are involved in the pathomechanism of ASD. Unlike the results of metal tests, changes in expression of neurotransmitter receptor genes in the same brain regions in both ASD models showed high similarities between the two groups. Full agreement of the direction of expression changes in the same brain regions in the VPA and THAL groups was observed in the case of 10 genes encoding proteins of all four types of receptors. For example, consistent reduction in the expression of the cholinergic genes *Chrm1, Chrm4*, and *Chrna7* and the dopaminergic gene *Drd1a* in CE of rats from both experimental groups is noteworthy. The same applies to an increase in *Chrna7* expression and a decrease in *Chrne* in the frontal cortex, as well as a decrease in expression of the same serotoninergic *Htr1b, Htr1d*, and *Htr3a* genes selectively in the HPC of rats from both groups.

Incorrect functioning of cholinergic receptors is considered as a potential pathomechanism in ASD (Deutsch et al., 2015; Gillentine and Schaaf, 2015; Wang et al., 2015; Marotta et al., 2020). Our results of examining the expression of genes coding for cholinergic receptor proteins in brains of rats in both ASD models in many cases show both agreements between experimental groups and similarity to clinical observations. A good example is a decline in the expression of the α7-nicotinic receptor gene, described in patients with various psychoneurological diseases, including ASD (Baranowska and Wisniewska, 2017; De Jaco et al., 2017; Hellmer and Nystrom, 2017). Additionally, the therapeutic effectiveness agonists of this receptor has been demonstrated (Ornoy, 2009; Lewis et al., 2018). The same was observed in studies on the transgenic mice showing, in addition to ASD-like behavioral symptoms, a significant reduction in the brain expression of several cholinergic receptor subunits, including α7 (Wang et al., 2016). Moreover, acetylcholinesterase inhibitor potentially increasing cholinergic activation, ameliorated repetitive compulsive behaviors in the VPA-induced mouse model of ASD (Eissa et al., 2019). Our results are therefore partly consistent with data indicating a reduction in cholinergic neurotransmission activity in rodents in the VPA-induced experimental model.

The results of our present study are also mostly consistent with the literature given above concerning ASD patients and show agreement between both rat ASD models. With few exceptions, we observed a reduction in cholinergic receptor expression, including the *Chrna7* gene encoding the α7 in both ASD models in both the CC and CE. The exception is the increase in the expression of the *Chrna7* gene encoding the α7 subunit of the nicotine receptor in the CC, observed in both ASD models, but particularly high in the VPA model (Tables 1, 2). This may be a compensatory effect, caused by a decrease in the level of protein and/or low activity of this receptor which was shown in the VPA induced mice model of ASD (Eissa et al., 2019).

The mechanisms of behavioral disorders in ASD are also linked to irregularities in the functioning of other neurotransmitter systems. The decrease in the activity of serotonergic receptors, both of patients and ASD animal models, is well documented (Muller et al., 2016; Saitow et al., 2020). Post-mortem studies have shown an age-related decrease in the 5-HT receptor density in the brains of autistic patients compared to controls (Oblak et al., 2013; Brandenburg and Blatt, 2019). There are also hypotheses according to which disorders of dopaminergic system receptor activity may underlie the development of behavioral disorders in patients with ASD (Staal et al., 2012, 2015; Gadow et al., 2014), as well as in animal models of ASD (Li et al., 2015; Liu et al., 2017; Brumback et al., 2018). In our studies in both ASD models, we found a decrease in mRNA levels in two dopaminergic genes. A similar effect was previously observed in the rat prefrontal cortex in the VPA model (Hara et al., 2015). It seems that also the disturbed balance in adrenergic receptor activity may promote the development of ASD. An increased number of autistic births has been reported in women who used beta2 receptor agonists during pregnancy (Gidaya et al., 2016; Su et al., 2017). In contrast, the administration of beta2 receptor antagonists to patients with ASD improved brain function as seen in fMRI studies (Hegarty et al., 2017; Sagar-Ouriaghli et al., 2018). In our studies presented in this work, both rat models of autism are dominated by the reduction of expression of genes encoding receptor proteins belonging to all the above-mentioned neurotransmitter systems. This suggests neurotransmission disorders, which is consistent with the results of studies on patients with ASD. The initial study of gene expression only at the mRNA level, the results of which are presented in this paper, aimed at identifying the most promising subjects for further detailed research. We plan to use these results in the next project, where we envisage a comprehensive study of the expression of selected genes at the mRNA, protein and, if possible, functional level.

In this work, the hypothetical interaction between Zn and Cu homeostasis disorders and neurotransmission in the brain in ASD is a link between the metallomics part and studies on the expression of genes encoding receptor proteins. There are numerous data from the literature indicating such connections. There is a hypothesis suggesting the environmental mechanism of reducing α7 receptor activity in ASD (Almeida et al., 2017), which may secondarily cause Zn deficiency as a compensatory mechanism to counteract α7 hypofunction (Fluegge Ba, 2017). On the other hand, it is known from the literature that Zn at micromolar concentrations enhances the activity of neuronal nicotinic receptors for acetylcholine even at saturating agonist concentrations, without changing the rate of receptor desensitization (Hsiao et al., 2008). According to the latter data, an increase, not a decrease in Zn concentration, could therefore be part of the mechanism compensating for the decrease in α7 activity. It is also known that Zn ions in low concentrations activate the serotonin receptors (Satala et al., 2018). In the rats, chronic Zn treatment induced increases in both 5-HT1AR protein levels and density of 5-HT1A receptor binding sites and also increased tissue levels of serotonin metabolite and its turnover (Szewczyk et al., 2017). Interactions between Zn ions and dopaminergic receptors and their hypothetical relationship with ASD are known (Hamilton et al., 2015). Besides, Zn modulates dopamine transport depending on concentration (Hamilton et al., 2015). Zn has previously been reported to act as a positive allosteric modulator of the beta (2) –adrenoceptor (Swaminath et al., 2002). Others have shown that Zn and Cu at low micromolar concentrations have an affinity for cloned human α (1A) adrenoceptors (Ciolek et al., 2011). *In vivo* studies in mice supplemented with Zn showed an increase in beta-adrenoceptor density and a decrease in alpha (1)-adrenoceptor density in the cerebral cortex, while receptor affinity did not change significantly (Viticchi et al., 1999).

In our ASD animal models, a decrease in the mRNA levels predominates in changes in the expression of tested genes of the selected receptors. In contrast, the concentrations of Zn and Cu increase, which mainly concerns the THAL model. Although no receptors protein levels or their activity were determined in this screening study, one can hypothesize that accumulation of Zn in the brain of rats in the THAL model may be a compensatory mechanism, a response to a receptors dysfunction. This compensatory nature can also be attributed to some of the results of our previous metabolomic studies (Toczylowska et al., 2020) in which in both rat ASD models in the hippocampus we detected an increase in hypoxanthine levels, which can lead to an increase in serotonergic receptor activity (Torres and Puig, 2015).

## Conclusion

This sum up, in contrast to the data obtained in studies with ASD patients, in our studies on rat models of autism induced by VPA or THAL, we did not find a decrease in zinc content or an increase in serum copper concentration. However, in the THAL model, we found an increase in the concentration of Zn and Cu in the serum and selected areas of the brain. These differences in comparison with the results of studies on patients, and the significant differences in the results between both rat models of ASD, may indicate the low significance and probably secondary contribution of Zn and Cu homeostasis disorders in the pathomechanism of behavioral disorders in ASD. In contrast, in both experimental groups, in the same areas of the brain, we found very similar disorders, mainly a decrease, in the expression of genes encoding receptor proteins for several neurotransmitters. These changes suggest neurotransmission disorders, also described in patients with ASD. Although the results obtained did not reveal clear interrelationships between Zn and Cu concentration disorders and changes in gene expression for neurotransmitters, we hypothesize based on the literature that an increase in Zn content in the brain in the THAL model may be part of the mechanism compensating for a dysfunction of neurotransmitter receptors. The results of our metallomic and genes expression studies on rat ASD models induced by VPA or THAL application *in utero*, described in the present manuscript, as well as previously published metabolomic profiles, allow for a number of working hypotheses that could be verified using using transgenic animal models of ASD (Patel et al., 2018; Rylaarsdam and Guemez-Gamboa, 2019). We anticipate carrying out such studies in the future.

## Data Availability Statement

The raw data supporting the conclusions of this article will be made available by the authors, without undue reservation.

## Ethics Statement

The animal study was reviewed and approved by Fourth Local Ethical Committee in Warsaw, resolution no. 43/2015 on May 22, 2015.

## Author Contributions

EZ, JA, and JL performed the conceptualization. EZ, JA, and AR performed the methodology and investigation. BT, JA, AR, and EZ performed the formal analysis and wrote original draft. JL wrote, reviewed, and edited the manuscript and performed the supervision. All authors have read and agreed to the published version of the manuscript.

## Conflict of Interest

The authors declare that the research was conducted in the absence of any commercial or financial relationships that could be construed as a potential conflict of interest.

## Footnotes

1 http://www.ncbi.nlm.nih.gov/tools/primer-blast/
