# Supplementary material for: Zinc and Copper Brain Levels and Expression of Neurotransmitter Receptors in Two Rat ASD Models
Source: Front Mol Neurosci. 2021 Jun 29;14:656740. doi: 10.3389/fnmol.2021.656740 (PMC8277171; doi:10.3389/fnmol.2021.656740)
Supplement: Supplementary file 1 [file Data_Sheet_1.docx]

**Genes belonging to the dopamine, serotonin, acetylcholine, and adrenergic receptor signaling pathways whose expression has been studied in this project**

The lists of the dopamine, serotonin, acetylcholine, and adrenergic receptor signalling pathways genes examined in the present study are presented on Table S1.

| ***Lp.*** | ***Gene symbol*** | ***Gene name*** |
| --- | --- | --- |
|  | *Adcy7* | Adenylate Cyclase 7 |
|  | *Adra1a* | Adrenoceptor Alpha 1A |
|  | *Adra1d* | Adrenoceptor Alpha 1D |
|  | *Adra2a* | Adrenoceptor Alpha 2A |
|  | *Adrb3* | Adrenoceptor Beta 3 |
|  | *Adrbk1/Grk2* | G Protein-Coupled Receptor Kinase 2 |
|  | *Chrm1* | Cholinergic Receptor Muscarinic 1 |
|  | *Chrm4* | Cholinergic Receptor Muscarinic 4 |
|  | *Chrm5* | Cholinergic Receptor Muscarinic 5 |
|  | *Chrna3* | Cholinergic Receptor Nicotinic Alpha 3 Subunit |
|  | *Chrna4* | Cholinergic Receptor Nicotinic Alpha 4 Subunit |
|  | *Chrna5* | Cholinergic Receptor Nicotinic Alpha 5 Subunit |
|  | *Chrna6* | Cholinergic Receptor Nicotinic Alpha 6 Subunit |
|  | *Chrna7* | Cholinergic Receptor Nicotinic Alpha 7 Subunit |
|  | *Chrne* | Cholinergic Receptor Nicotinic Epsilon Subunit |
|  | *Drd1* | Dopamine Receptor D1 |
|  | *Drd2* | Dopamine Receptor D2 |
|  | *Drd5* | Dopamine Receptor D5 |
|  | *Htr1a* | 5-Hydroxytryptamine (Serotonin) Receptor 1A |
|  | *Htr1b* | 5-Hydroxytryptamine (Serotonin) Receptor 1B |
|  | *Htr1d* | 5-Hydroxytryptamine (Serotonin) Receptor 1D |
|  | *Htr1f* | 5-Hydroxytryptamine (Serotonin) Receptor 1F |
|  | *Htr2a* | 5-Hydroxytryptamine (Serotonin) Receptor 2A |
|  | *Htr3a* | 5-Hydroxytryptamine (Serotonin) Receptor 3A |
|  | *Htr4* | 5-Hydroxytryptamine (Serotonin) Receptor 4 |
|  | *Htr7* | 5-Hydroxytryptamine (Serotonin) Receptor 7 (Adenylate Cyclase-Coupled) |

Table S1. Genes essential for dopamine, serotonin, acetylcholine and adrenergic receptor signaling pathways tested in these studies.

To better understand the molecular role that genes selected in this project play in ASD pathogenesis, an analysis of the predicted function of these genes was performed. The studied genes were divided into categories, taking into account the following function: behavior, learning, memory, cognition, metal ion homeostasis, divalent metal ion transport, metal ion sequestration. The interactions between all analyzed genes obtained in the *Genemania* (<https://genemania.org/>) are presented in Figure S1.


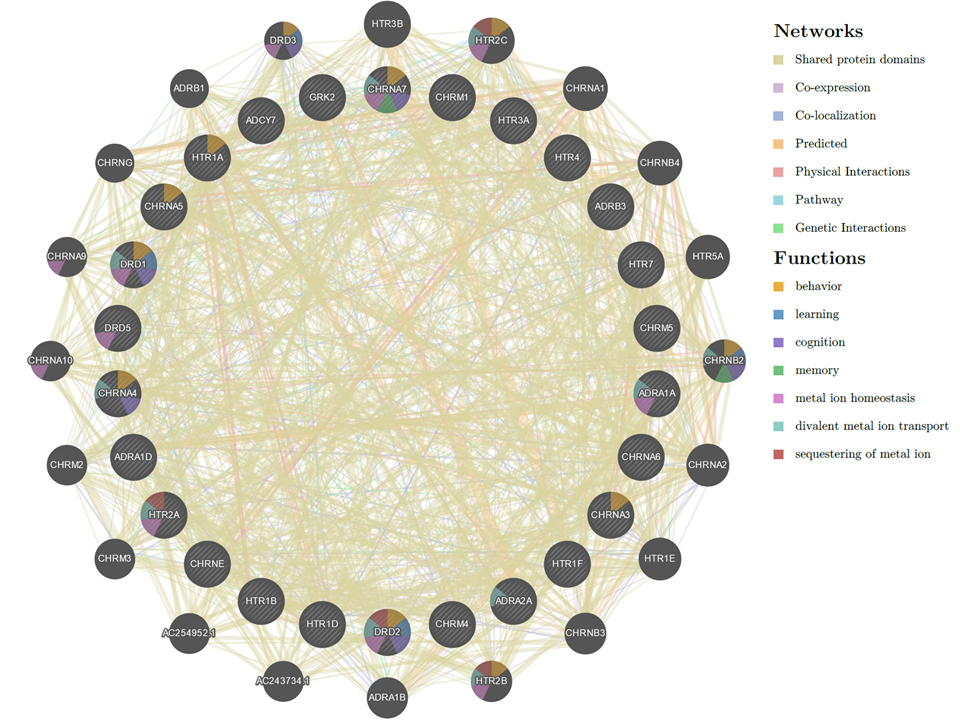


Figure S1. The Cytoscape presents a set of genes involved in the **dopamine, serotonin, acetylcholine,** and **adrenergic receptor signaling pathways**. The *in silico* network shows the interactions between selected genes tested in this study, which are essential for the regulation of **behavior, learning, cognition**, and **memory.** Their potential role in the **metal ion homeostasis, divalent metal ion transport,** and **sequestering of the metal ion** is also presented.

In the *in silico* studies of 26 genes (Figure S1.), we showed that *CHRNA7, CHRNA5, HTR1A, DRD1, CHRNA4, DRD2, CHRNA3* genes are responsible for **behavior,** *DRD1*, and *DRD2* are involved in the **learning** process regulation, *CHRNA7, DRD2, CHRNA4, DRD1* participate in the **cognition** function regulation. We showed also that *CHRNA7* plays an important role in the regulation process of **memory**. The analysis also confirmed the important functions of selected genes in **metal ion homeostasis** (*CHRNA7, ADRA1A, DRD2, HTR2A, DRD5, DRD1*), **divalent metal ion transport** (*CHRNA7, ADRDA1A, DRD2, HTR2A, ADRA2A, CHRNA4, DRD1*) and **sequestering of metal ion** (*HTR2A, DRD2*). In our network, 51.69% of the protein encoded by the analyzed genes shared protein domains, 14.05% is co-expressed, 11.27% share co-localization, 7.25% is in physical interaction, 6.30% share signaling pathway and 0.14% - genetic interactions. Based on bioinformatics analyzes, the role in this network is predicted in 9.29% of gens.

The analyzed sets of genes were divided into four groups of genes belonged to the **dopamine, serotonin, acetylcholine,** and **adrenergic receptor signaling pathways**. The molecular functions of these groups of genes have been analyzed in detail.

The analysis showed that in the *DRD1, DRD2, DRD5* genes are responsible for the regulation of dopamine receptor signaling, dopaminergic synaptic transmission, G protein-coupled receptor signaling pathway coupled to the cyclic nucleotide second messenger, but also for **cellular metal ion homeostasis.**


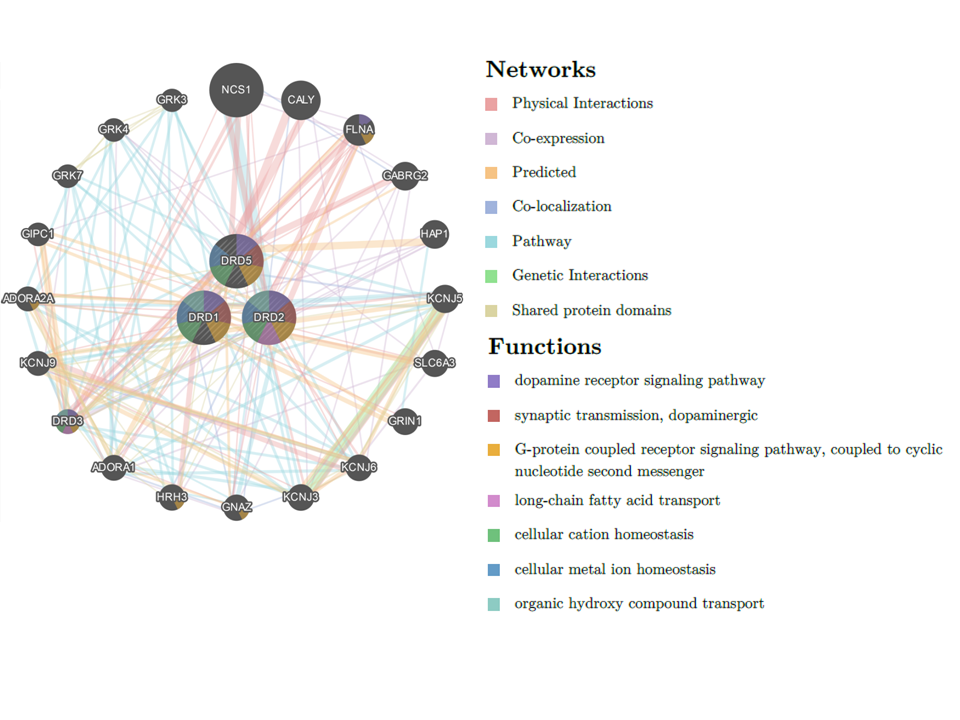


Figure S2. *In silico* network~~s~~ modeling shows predicted functions and interactions in the analyzed genes belonging to the **dopamine receptor signaling pathways**

Moreover, *DRD1* and *DRD2* perform a function in the transport of the organic hydroxyl compounds. In addition, the *DRD2* gene is involved in the transport of long chain fatty acids. The genes involved in this network share: physical interactions in 67.64%; co-expression in 13.50%; prediction in action (based on bioinformatics analyzes) in 6.35%; co-localization in 6.17%; signaling pathways in 4.35%; genetic interaction (on mRNA or DNA level) in 1.4% and protein domains in 0.59%. (Figure S2).

Analysis indicates that *HTR1A, HTR4, HTR1F, HTR1B, HTR7, HTR3A, HTR2A* are important for serotonin receptor signaling pathway, *HTR1A, HTR4, HTR1F, HTR1B, HTR1D, HTR3A, HTR2A* for G-protein coupled serotonin receptor activity, while *HTR2A* participates in the sequestering of calcium ion and metal ion. The genes involved in this network share: protein domain in 53.18%; physical interactions in 30.90%; prediction in action (based on bioinformatics analyzes) in 6.02%; co-localization in 5.56%; co-expression in 4.10% and genetic interaction (on mRNA or DNA level) in 0.25% (Figure S3).


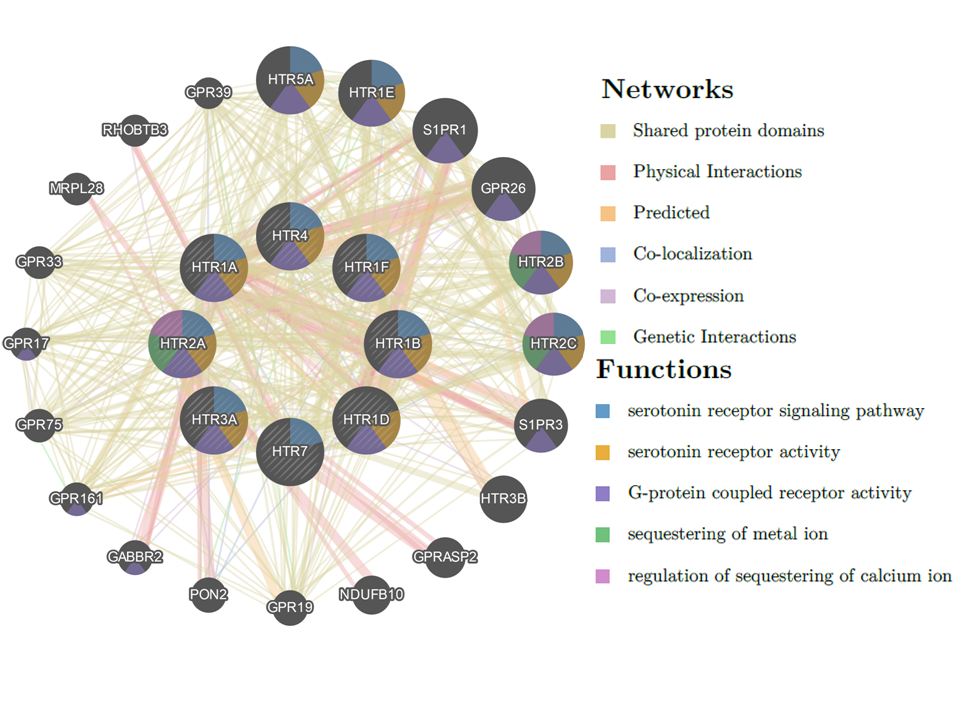


Figure S3. *In silico* network~~s~~ modeling shows predicted functions and interactions in the analyzed genes belonging to the **serotonin receptor signaling pathways**

The following genes have been classified as involved in acetylcholine receptor activity: *CHRNA4, CHRNE, CHRNA5, CHRNA6, CHRNA3, CHRNA7*. The *CHRNA4, CHRNE, CHRNA5, CHRNA7, CHRNA3* genes take part in the creation of acetylcholine-gated channel complex, *CHRNA4, CHRNA3, CHRNE* are involved in the cholinergic synaptic transmission, while *CHRM1, CHRM4, CHRM5* participate in the G-protein coupled acetylcholine receptor signaling pathway. Moreover, *CHRNA4, CHRNA3* participate in the amino transport and *CHRNA4* in the action potential regulation. The genes involved in this network share: protein domain in 71.82%; prediction in action (based on bioinformatics analyzes) in 16.69%; co-expression in 11.49% (Figure S4).


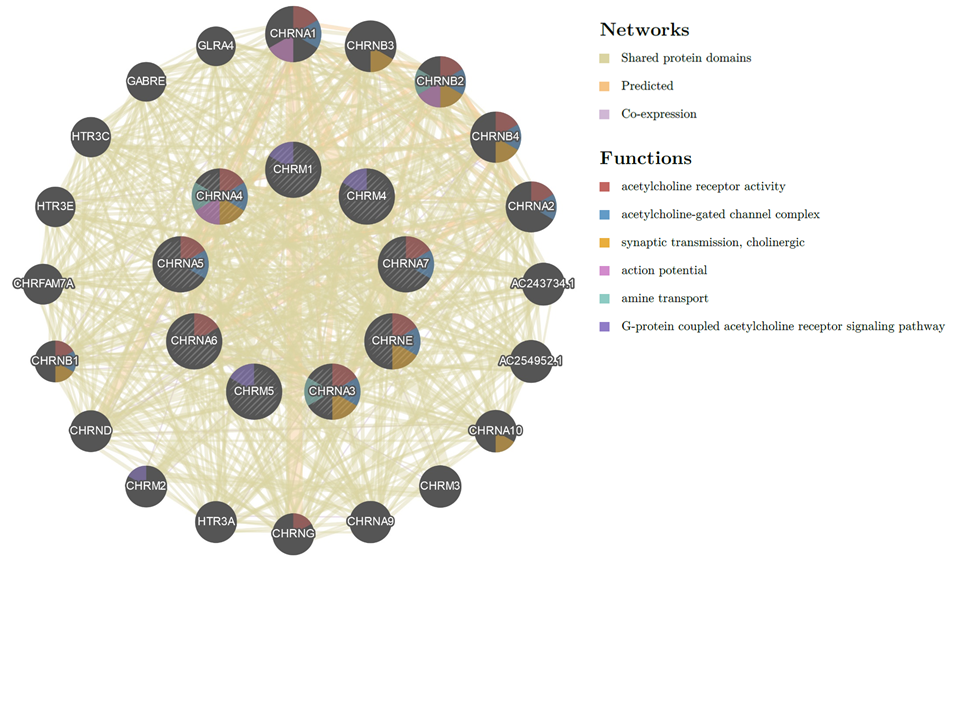


Figure S4. *In silico* network~~s~~ modeling shows predicted functions and interactions in the analyzed genes belonging to the **acetylcholine receptor signaling pathways**

The last group of analyzed genes play an important function in the adenylate cyclase-modulating G-protein coupled receptor signaling pathway (*ADRA2A, ADRA1D, ADRB3, ADCY7*), adrenergic receptor signaling pathway (*ADRA2A, ADRA1D, ADRB3, ADRA1A*), and positive regulation of phospholipase activity (*GRK2, ADCY7, ADRA1A*). The analysis also showed that genes belonging to this group are involved in the regulation of ion transmembrane transport (*ADRA2A*), calcium ion transport (*ADRA1A, ADRA2A*), **cellular metal ion homeostasis** (*ADRA1A*) and **divalent metal ion transport** (*ADRA1A, ADRA1A*). The genes involved in this network share: prediction in action (based on bioinformatics analyzes) in 50.57%; protein domain in 30.63%; signaling pathways in 7.51%; physical interactions in 5.21%; co-expression in 3.61%; co-localization in 2.05%; and genetic interaction (on mRNA or DNA level) in 0. 0.42% (Figure S5).


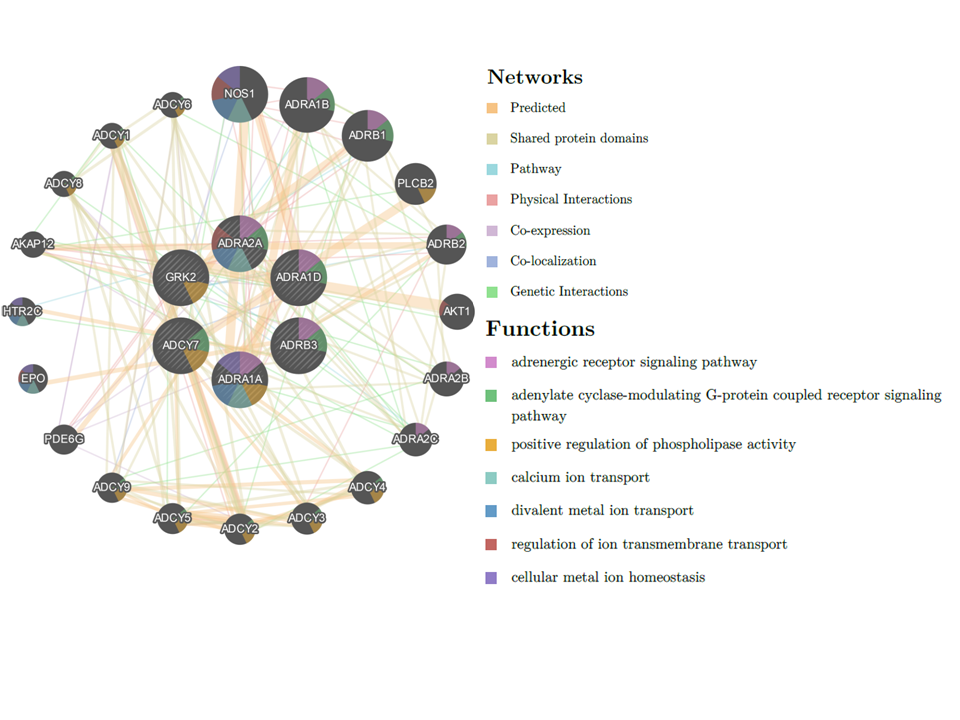


Figure S5. *In silico* network~~s~~ modeling shows predicted functions and interactions in the analyzed genes belonging to the **adrenergic receptor signaling pathways**

In this project, we have shown that different brain structures are characterized by different changes in gene expression associated with the exposure of rats to THAL or VPA. We did not detect genes in which expression changes would be the same in all structures at once (Figure S6).

In this project, we showed that different brain structures were characterized by different changes in gene expression associated with the exposure of rats to THAL or VPA. We did not detect genes in which expression changes would be the same in all structures at once (Figure S6). In rats treated with THAL 6 genes were down-regulated in the CC (*Drd5*, *Adrb3, Adrbk1, Chrm5, Drd2, Chrne*); 7 in the CE (*Adra1d, Chrm1, Chrm4, Chrna4, Chrna7, Htr1f, Drd1a*); 3 in the HPC (*Htr1b, Htr1d, Htr3a*). After THAL exposition only 2 genes in rats CC were up-regulated (*Adra2a, Chrna7*). In rats treated with VPA down-regulated were 2 genes at the CC (*Drd2, Chrne*), 8 genes in the CE (*Adcy7, Adra1a, Adra1d, Chrm1, Chrm4, Chrna5, Chrna7, Drd1a*) and 3 in the HPC (*Htr1b, Htr1d, Htr3a*), while up-regulated were 4 genes (*Adra1d, Adra2a, Adrb3, Chrna5*). These results are presented in Table 1 and 2 in the main paper.


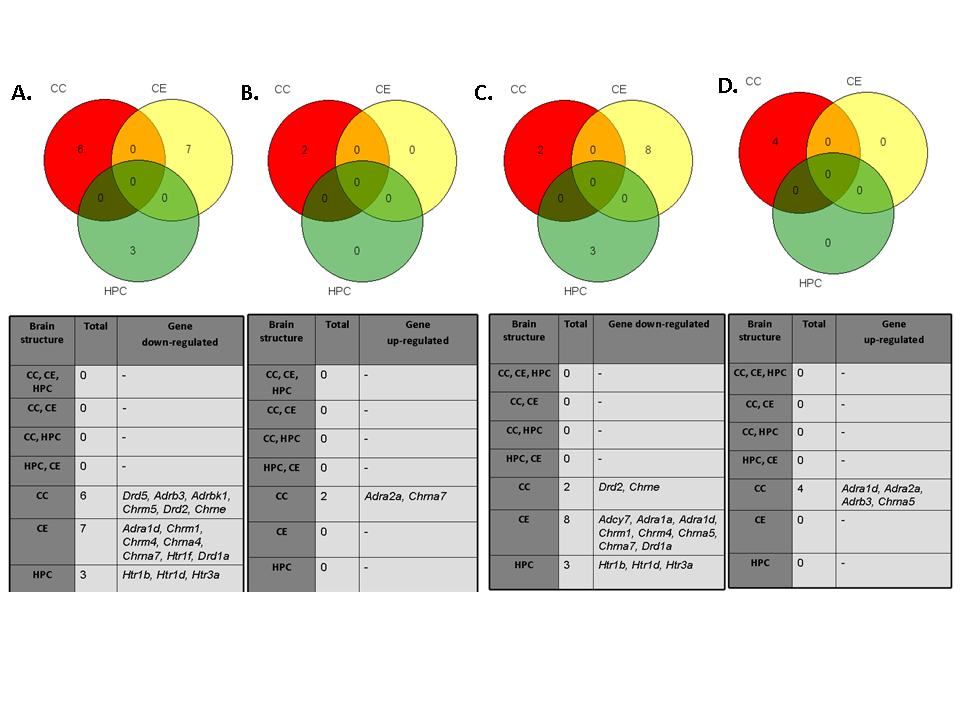


Figure S6. Venn diagram of differentially expressed dopamine, serotonin, acetylcholine and adrenergic receptor signalling pathways genes in the cerebral cortex (CC), cerebellum (CE) and hippocampus (HPC) of THAL or VPA-treated rats. (A) Genes down-regulated in the different brain structure of THAL-treated rats; (B) Genes up-regulated in the different brain structure of THAL-treated rats; (C) Genes down-regulated in the different brain structure of VPA-treated rats; (D) Genes up-regulated in the different brain structure of VPA-treated rats

We used two recognized genetic online databases to assess the relationship of the studied genes to the pathogenesis of autism spectrum diseases (ASD): The Simons Foundation Autism Research Initiative (SFARI) gene database (<https://gene.sfari.org/>) (Table S2) and Autism database (AUTDB) (<http://autism.mindspec.org/>) (Table S3). Taking into account the SFARI Human Gene database criteria (<https://gene.sfari.org/about-gene-scoring/criteria/>) and Autism AUTDB (<http://autism.mindspec.org/>), we presented the relationship between the ASD genes we analyzed. Details of the relationship of studied genes connected with ASD from SFARI and AUTDB were presented in the Table S2 and Table S3 respectively.

| ***Gene*** | ***Name*** | ***Alias*** | ***Associated disorders*** | ***Score*** | ***Molecular function*** | ***Relevance to autism*** |
| --- | --- | --- | --- | --- | --- | --- |
| *DRD1* | Dopamine Receptor D1 | *DRD1, DRDR, DRD1A* | - | 3. (Suggestive evidence) | This gene encodes the D1 subtype of the dopamine receptor, the most abundant dopamine receptor in the central nervous system. This G-protein coupled receptor stimulates adenylyl cyclase and activates cyclic AMP-dependent protein kinases. D1 receptors regulate neuronal growth and development, mediate some behavioral responses, and modulate dopamine receptor D2-mediated events. | A *DRD1* haplotype was found to be associated with risk for autism spectrum disorders in male-only affected sib-pair families (Hettinger et al., 2008). |
| *DRD2* | Dopamine Receptor D2 | *DRD2, D2DR, D2R* | Tourette syndrome | 3. (Suggestive evidence) | This gene encodes the D2 subtype of the dopamine receptor. This G-protein coupled receptor inhibits adenylyl cyclase activity. A missense mutation in this gene causes myoclonus dystonia; other mutations have been associated with schizophrenia. | Case-control and family-based association analysis of the *DRD2* gene in a cohort of 112 male-only affected sib-pair ASD families and a comparison cohort of 253 controls revealed an increased frequency of the rs1800498 TT genotype in affected males compared to the comparison group (p=0.007) (Hettinger et al., 2012). Family-based assocation tests in the same report showed that the rs1800498 T allele was over-transmitted to affected males (p=0.0003) under an additive model. |
| *CHRNA7* | Cholinergic Receptor Nicotinic Alpha 7 Subunit | *CHRNA7, CHRNA7-2, NACHRA7* | ASD, ID, EPS, DD/NDD, BPD, EP | 2.  (Strong Candidate) | The nicotinic acetylcholine receptors (nAChRs) are members of a superfamily of ligand-gated ion channels that mediate fast signal transmission at synapses. The nAChRs are thought to be hetero-pentamers composed of homologous subunits. The protein encoded by this gene forms a homo-oligomeric channel, displays marked permeability to calcium ions and is a major component of brain nicotinic receptors that are blocked by, and highly sensitive to, alpha-bungarotoxin. Once this receptor binds acetylcholine, it undergoes an extensive change in conformation that affects all subunits and leads to opening of an ion-conducting channel across the plasma membrane. | A rare deletion in the *CHRNA7* gene has been identified with developmental delay and intellectual disability (Mikhail et al., 2011). In addition, a rare *CHRNA7* duplication was found in two patients with autism and intellectual disability (Leblond et al., 2012). |
| *HTR1B* | 5-Hydroxytryptamine (Serotonin) Receptor 1B | *HTR1B, S12,*  *5-HT1B, HTR1D2, HTR1DB, 5-HT1DB, HTR1B* | - | 3. (Sugegestive evidence) | The encoded protein belongs to G-protein coupled receptor 1 family. | Genetic association has been found between the *HTR1B* gene and autism in a Brazilian population cohort (Orabona et al., 2009). |
| *HTR3A* | 5-Hydroxytryptamine (Serotonin) Receptor 3A | - | - | 3. (Suggestive evidence) | ligand-gated ion channel receptor | Genetic association has been found between the *HTR3A* gene and autism in a Caucasian-American population cohort (Anderson et al., 2009). In addition, genetic association has been found between *HTR3A* and therapeutic response to risperidone treatment in Chinese schizophrenic patients. |

Table S2. Analyzed genes associated with ASD. The Simons Foundation Autism Research Initiative (SFARI) database

References:

1. Hettinger JA, Liu X, Schwartz CE, Michaelis RC, Holden JJ., A DRD1 haplotype is associated with risk for autism spectrum disorders in male-only affected sib-pair families., Am J Med Genet B Neuropsychiatr Genet. 2008 Jul 5;147B(5):628-36. doi: 10.1002/ajmg.b.30655.
2. Hettinger JA, Liu X, Hudson ML, Lee A, Cohen IL, Michaelis RC, Schwartz CE, Lewis SM, Holden JJ., DRD2 and PPP1R1B (DARPP-32) polymorphisms independently confer increased risk for autism spectrum disorders and additively predict affected status in male-only affected sib-pair families., Behav Brain Funct. 2012 May 4;8:19. doi: 10.1186/1744-9081-8-19.
3. Mikhail FM, Lose EJ, Robin NH, Descartes MD, Rutledge KD, Rutledge SL, Korf BR, Carroll AJ., Clinically relevant single gene or intragenic deletions encompassing critical neurodevelopmental genes in patients with developmental delay, mental retardation, and/or autism spectrum disorders., Am J Med Genet A. 2011 Oct;155A(10):2386-96. doi: 10.1002/ajmg.a.34177.
4. Leblond CS, Heinrich J, Delorme R, Proepper C, Betancur C, Huguet G, Konyukh M, Chaste P, Ey E, Rastam M, Anckarsäter H, Nygren G, Gillberg IC, Melke J, Toro R, Regnault B, Fauchereau F, Mercati O, Lemière N, Skuse D, Poot M, Holt R, Monaco AP, Järvelä I, Kantojärvi K, Vanhala R, Curran S, Collier DA, Bolton P, Chiocchetti A, Klauck SM, Poustka F, Freitag CM, Waltes R, Kopp M, Duketis E, Bacchelli E, Minopoli F, Ruta L, Battaglia A, Mazzone L, Maestrini E, Sequeira AF, Oliveira B, Vicente A, Oliveira G, Pinto D, Scherer SW, Zelenika D, Delepine M, Lathrop M, Bonneau D, Guinchat V, Devillard F, Assouline B, Mouren MC, Leboyer M, Gillberg C, Boeckers TM, Bourgeron T., Genetic and functional analyses of SHANK2 mutations suggest a multiple hit model of autism spectrum disorders., PLoS Genet. 2012 Feb;8(2):e1002521. doi: 10.1371/journal.pgen.1002521. Epub 2012 Feb 9.
5. Orabona GM, Griesi-Oliveira K, Vadasz E, Bulcão VL, Takahashi VN, Moreira ES, Furia-Silva M, Ros-Melo AM, Dourado F, Matioli SR, Otto P, Passos-Bueno MR., HTR1B and HTR2C in autism spectrum disorders in Brazilian families., Brain Res. 2009 Jan 23;1250:14-9. doi: 10.1016/j.brainres.2008.11.007. Epub 2008 Nov 12.
6. Anderson BM, Schnetz-Boutaud NC, Bartlett J, Wotawa AM, Wright HH, Abramson RK, Cuccaro ML, Gilbert JR, Pericak-Vance MA, Haines JL., Examination of association of genes in the serotonin system to autism., Neurogenetics. 2009 Jul;10(3):209-16. doi: 10.1007/s10048-009-0171-7. Epub 2009 Jan 28.

| Unique ID | Gene | Name | Genetic category | # of variants | Associated disorders | # of reports | Primary reference |
| --- | --- | --- | --- | --- | --- | --- | --- |
| GEN292 | *CHRNA7* | Cholinergic receptor, nicotinic, alpha 7 | Multigenic CNV | 31 | ASD, ADHD, BPD, DD, EP, ID, SCZ | 13 | Mikhail, et. al., 2011 |
| GEN576 | *DRD1* | Dopamine receptor D1 | Genetic association | 4 | ASD, ADHD | 4 | Hettinger, et. al., 2008 |
| GEN358 | *DRD2* | Dopamine receptor D2 | Rare single gene variant | 8 | ASD, EP, SCZ | 6 | Hettinger, et. al., 2012 |
| GEN127 | *HTR1B* | 5-hydroxytryptamine  (serotonin) receptor 1A | Genetic association | 2 | ASD, EP | 6 | Orabona, et. al., 2008 |
| GEN128 | *HTR2A* | 5-hydroxytryptamine  (serotonin) receptor 2A | Genetic association | 12 | ASD, EP | 10 | Veenstra-VanderWeele, etal., 2002 |
| GEN129 | *HTR3A* | 5-hydroxytryptamine  (serotonin) receptor 3A | Functional | 5 | ASD, EP, SCZ | 7 | Anderson, et. al., 2009 |
| GEN131 | *HTR7* | 5-hydroxytryptamine  (serotonin) receptor 7 (adenylate cyclase-coupled) | Genetic association | 6 | ASD, BPD, EP, SCZ | 5 | Lassing, et. al., 1999 |

Table S3. Analyzed genes associated with ASD. The Autism database (AUTDB).

References:

1. Mikhail FM, Lose EJ, Robin NH, Descartes MD, Rutledge KD, Rutledge SL, Korf BR, Carroll AJ., Clinically relevant single gene or intragenic deletions encompassing critical neurodevelopmental genes in patients with developmental delay, mental retardation, and/or autism spectrum disorders., Am J Med Genet A. 2011 Oct;155A(10):2386-96. doi: 10.1002/ajmg.a.34177.
2. Hettinger JA, Liu X, Schwartz CE, Michaelis RC, Holden JJ., A DRD1 haplotype is associated with risk for autism spectrum disorders in male-only affected sib-pair families., Am J Med Genet B Neuropsychiatr Genet. 2008 Jul 5;147B(5):628-36. doi: 10.1002/ajmg.b.30655.
3. Hettinger JA, Liu X, Hudson ML, Lee A, Cohen IL, Michaelis RC, Schwartz CE, Lewis SM, Holden JJ., DRD2 and PPP1R1B (DARPP-32) polymorphisms independently confer increased risk for autism spectrum disorders and additively predict affected status in male-only affected sib-pair families., Behav Brain Funct. 2012 May 4;8:19. doi: 10.1186/1744-9081-8-19.
4. Orabona GM1, Griesi-Oliveira K, Vadasz E, Bulcão VL, Takahashi VN, Moreira ES, Furia-Silva M, Ros-Melo AM, Dourado F, Matioli SR, Otto P, Passos-Bueno MR., HTR1B and HTR2C in autism spectrum disorders in Brazilian families., Brain Res. 2009 Jan 23;1250:14-9. doi: 10.1016/j.brainres.2008.11.007. Epub 2008 Nov 12.
5. Veenstra-VanderWeele J, Kim SJ, Lord C, Courchesne R, Akshoomoff N, Leventhal BL, Courchesne E, Cook EH Jr., Transmission disequilibrium studies of the serotonin 5-HT2A receptor gene (HTR2A) in autism., Am J Med Genet. 2002 Apr 8;114(3):277-83.
6. Anderson BM1, Schnetz-Boutaud NC, Bartlett J, Wotawa AM, Wright HH, Abramson RK, Cuccaro ML, Gilbert JR, Pericak-Vance MA, Haines JL., Examination of association of genes in the serotonin system to autism., Neurogenetics. 2009 Jul;10(3):209-16. doi: 10.1007/s10048-009-0171-7. Epub 2009 Jan 28.
